# Prevalence of Transfusion-Transmitted Infections and Its Association With Blood Groups Among Blood Donors at the Blood Bank of a Tertiary Care Center

**DOI:** 10.7759/cureus.86626

**Published:** 2025-06-23

**Authors:** Qurana Khatoon, Sayeedul Hasan Arif

**Affiliations:** 1 Pathology, Madhubani Medical College and Hospital, Madhubani, IND; 2 Pathology, Jawaharlal Nehru Medical College, Aligarh Muslim University, Aligarh, IND

**Keywords:** blood bank, blood donors, blood group antigens, blood groups, transfusion-transmitted infections

## Abstract

Background

Safe blood transfusion remains a challenge, and transmission of infections is always a concern. The detection of infectious diseases in blood donors ensures the safety of blood transfusion. Another challenge is the differences in blood group antigens due to the ability of clinically significant antibodies to cause adverse reactions. This study aimed to evaluate the pattern of distribution and the possible correlation of transfusion-transmitted infections (TTIs) with different blood groups. This study also aimed to evaluate the frequencies of minor blood group antigens detected on screening in a few infected blood donors.

Methodology

This study included replacement donors who were fit to donate blood after screening. After blood donation, tests for TTIs was done using the chemiluminescence immunoassay for hepatitis B virus (HBV)/human immunodeficiency virus (HIV)/hepatitis B virus (HCV), and rapid diagnostic kits were used for malaria and syphilis. Blood grouping was performed by IMMUCOR’S NEO IRIS instrument using commercial antisera. Samples of blood donors infected with TTIs over the study period of two months were also tested for minor antigens. Statistical analysis was done using SPSS Software, version 20 (IBM Corp., Armonk, NY, USA).

Results

In total, 46,549 blood donors were included, of whom 3,064 (6.6%) donors were reactive for TTIs, with the most prevalent being HBV infection (n = 1,183, 2.5%). In this study, 2.4% (n = 23) of the total B-negative blood donors were HCV reactive (χ^2^ = 4.3, p = 0.038; statistically significant), and 0.9% (n = 8) of the total B-negative blood donors were malaria reactive (Fisher’s exact test = 11.06, p = 0.001; statistically significant). Blood donors with A-positive blood group were significantly associated with syphilis infection (χ^2^ = 3.86, p = 0.05). The Fyb antigen was relatively more prevalent among HIV-reactive donors (n = 24, 77.4%), while E (n = 3, 9.7%) and c (n = 13, 41.9%) antigens were relatively lesser prevalent compared to other infected donors. Similarly, malaria-reactive donors had a lower prevalence of C antigen (n = 17, 73.9%) and a higher prevalence of E antigen (n = 8, 34.8%). The most common Rh phenotype found among all the TTI-infected reactive donors was CCDee, and among the Rh-negative infected donors, ccdee was the only phenotype observed.

Conclusions

This study determined the regional prevalence of TTIs among blood donors and noted a significant association of B-negative blood group with HCV and malaria. This study also assessed the frequency of some minor blood group antigens among TTI-infected blood donors.

## Introduction

Blood donation is one of the most significant contributions that a person can make to society. Blood can be stored for a limited period, which is why blood banks need a steady and constant supply. The International Society of Blood Transfusion (ISBT) established a Working Party in 1980 to devise a genetically based numerical terminology for red cell surface antigens [1]. According to the Red Cell Immunogenetics and Blood Group Terminology Working Party of ISBT, there are now 47 recognized blood group systems, and 52 genes responsible for these systems have been identified [2].

Blood centers have implemented education programs and screening procedures aimed at reducing the risk of transfusion-transmitted viral infections, and efforts are underway to improve behavioral screening of donors. Most infectious units are believed to be from donors who donate in the window period, namely, the time between infection and detectability by screening tests. Thus, there has been a continuous effort to develop more sensitive and specific screening tests for transfusion-transmitted infections (TTIs) among blood donors. Infectious disease detection in the blood donor population provides a mechanism to assess the safety of the blood supply and the effectiveness of donor deferral criteria and other screening measures.

As blood is a human product, transmission of infections is always a threat to blood safety. According to the Directorate General of Health Services guidelines for blood banks in India, each blood unit should be tested for human immunodeficiency virus (HIV) I and II antibodies, hepatitis B surface antigen (HBsAg), hepatitis C virus (HCV) antibody, Venereal Disease Research Laboratory tests, and malarial parasite [3].

Blood group antigens are proteins or carbohydrates and are the surface markers of the red cell membrane [4]. Knowledge of blood group antibodies helps in blood transfusion in patients with blood group antibodies that are not commonly encountered and for which antigen-negative blood is not available in the routine stock.

Blood transfusion is not only associated with transmission of infections, but clinically significant antibodies can also cause adverse reactions following transfusion, ranging from mild to severe, and cause hemolytic disease of the fetus and newborn due to placental transfer of red blood cells from the mother to the fetus. Decision on the clinical significance of an antibody when compatible blood is not immediately available is likely to remain one of the most common dilemmas facing transfusion practitioners [5].

The Hirszfelds discovered at the end of the First World War that the frequency of the ABO blood groups varied in different people [6]. The differences in blood group antigen distribution are important due to their influence on the clinical practice of transfusion medicine. Hence, this study aims to determine the prevalence of these infections among the blood donors in our blood bank and their possible association with blood group types. The first study objective was to evaluate the pattern of distribution and the possible correlation of TTIs with different blood groups. The second objective was to evaluate the frequencies of minor blood group antigens in a few infected blood donors detected on screening. This study found some minor blood group antigen frequencies among the transfusion-transmitted disease-infected blood donors.

## Materials and methods

Study design, population, and sample size

This study was conducted at the Blood Bank, Department of Pathology, Jawaharlal Nehru Medical College, Aligarh Muslim University, Aligarh, India, from January 2017 to June 2019. This study was retrospective from January 2017 to July 2018 and prospective from August 2018 to June 2019. All replacement blood donors were fit to donate blood as per the Standard Operating Procedures Manual of our Blood Bank.

Study measures

Blood grouping was performed by the US FDA-approved Immucor’s NEO Iris fully automated system [7,8]. Tests for TTIs were done using chemiluminescence immunoassay (VITROS ECiQ/3600 Immunodiagnostic system) for HBV/HIV/HCV, which uses the principle of enhanced chemiluminescence using MicroWell technology. Rapid plasma reagin card test was used for syphilis, whereas for malaria antigen, a rapid diagnostic kit from AlereTrueline was used.

Samples of blood donors reactive for TTIs on screening over two months from December 2018 to January 2019 were tested weekly for extended phenotyping (D, C, c, E, e, K, k, Fya, Fyb, and S antigens). This was performed using the US FDA-approved NEO Iris fully automated immunohematology analyzer (Immucor, Rodermark, Germany) that uses the microplate hemagglutination technique for typing with IgM monoclonal antiserum and Capture-R Select (solid-phase red cell adherence) for typing with polyclonal IgG antiserum. The D, C, c, E, e, and K antigens were typed using monoclonal antisera from Immucor derived from clones D175-2/TH28, MS24, MS33, MS258+MS80, MS16+MS21, and MS56, respectively. Donors typed as D negative were confirmed using an antiglobulin weak D test in an automated solid phase test using Novaclone anti-D (Immunocor, Rodermark, Germany), which contains IgG clone D415 in addition to IgM clone D175-2. Fya, Fyb, S, and k antigens were typed by commercially prepared polyclonal antisera (Immucor) using Capture-R Select.

Protocols and recommendations mentioned in the Standard Operating Procedures Manual of the Blood Bank were followed for donor screening.

Inclusion criteria

The study included individuals with clinical fitness to donate blood based on history and examination, aged between 18 and 60 years, weighing more than or equal to 45 kg, having a pulse rate and blood pressure within the normal limit for age with normal temperature, and having a hemoglobin level of 12.5 g/dL or more with no obvious signs and symptoms of any disease. We included individuals who provided consent to donate blood for research purposes.

Exclusion criteria

The study excluded individuals who were identified as professional donors, with a positive history of HBV/HCV/HIV. We excluded intravenous drug abusers, those suffering from any malignancy, endocrine disease, epilepsy, mental afflictions, coagulation defects, cardiovascular or cerebrovascular diseases, chronic diseases, bleeding disorders, diabetes mellitus dependent on insulin, or asthma. Individuals with a history of tattooing, ear piercing, accidental needle stick injury, rabies vaccination, or hepatitis B immunoglobulin injection within one year were also excluded. Those with a history of malaria/typhoid/dengue in the last six months, jaundice in the last 10 years, tuberculosis in the last five years, history of blood donation in the past three months, and medications that are potentially harmful to the recipient were not permitted to donate. Females were not accepted as donors during pregnancy, 12 months after full-term delivery, during lactation, six months after second and third-trimester abortion, and menorrhagia.

Statistical analysis

After data collection, a template was generated in an MS Excel (Microsoft Corp., Redmond, WA, USA) sheet. Descriptive analysis and frequency distribution were performed using SPSS Software, version 20 (IBM Corp., Armonk, NY, USA). Prevalence and categorical variables were expressed as percentages. Test of association between blood groups (ABO and Rh) and TTIs was calculated using the chi-square test or Fisher’s exact test as applicable. A p-value less than or equal to 0.05 was considered statistically significant.

Ethical considerations

This study was approved by the Institutional Ethical Review Committee of Jawaharlal Nehru Medical College, Aligarh Muslim University, Aligarh (approval number: 1016/FM; dated 13/07/2018).

## Results

The total number of blood donors studied was 46,549, of whom 3,064 donors (6.6% ) were seropositive. The most prevalent TTI was HBV (n = 1,183, 2.5%), followed by HCV (n = 815, 1.8%), syphilis (n = 657, 1.4%), HIV (n = 275, 0.6%), and malaria (n = 134, 0.3%) (Table 1).

**Table 1:** Prevalence of tranfusion-transmission infections among Rh-positive and Rh-negative blood groups. HBV: hepatitis B virus; HCV: hepatitis B virus; HIV: human immunodeficiency virus

Rh status	Number of donors	HBV	HIV	HCV	Syphilis	Malaria	Total (%)
Rh positive	43,981	1,112 (2.5%)	256 (0.6%)	760 (1.7%)	625 (1.4%)	122 (0.3%)	2,875 (6.5%)
Rh negative	2,568	71 (2.8%)	19 (0.7%)	55 (2.1%)	32 (1.2%)	12 (0.5%)	189 (7.4%)
Total	46,549	1,183 (2.5%)	275 (0.6%)	815 (1.8%)	657 (1.4%)	134 (0.3%)	3,064 (6.6%)

HBV infection was found to be the most common in O-negative blood donors (n = 24, 3.3%) (Tables 2, 3). However, this association was not significant (χ^2^ = 1.8, p = 0.18).

**Table 2:** Most common blood group type observed in infected donors. HBV: hepatitis B virus; HCV: hepatitis B virus; HIV: human immunodeficiency virus

Infections	Blood group prevalence, n (%)	P-value	Chi-square or Fisher’s exact test
HBV	O negative, 24 (3.3%)	0.18	1.8
HIV	O negative, 8 (1.1%)	0.08	2.98 (Fisher’s exact test)
HCV	B negative, 23 (2.4%)	0.038	4.3
Syphilis	A positive, 148 (1.4%)	0.05	3.86
Malaria	B negative, 8 (0.9%)	0.001	11.06 (Fisher’s exact test)

**Table 3:** Prevalence of transfusion-transmitted infections among different blood groups. HBV: hepatitis B virus; HCV: hepatitis B virus; HIV: human immunodeficiency virus

Blood group	Number of donors (%)	HBV	HIV	HCV	Syphilis	Malaria	Total (%)
A positive	10,534 (22.6%)	261 (2.5%)	55 (0.5%)	197 (1.9%)	148 (1.4%)	30 (0.3%)	691 (6.6%)
B positive	16,135 (34.7%)	409 (2.5%)	96 (0.6%)	253 (1.6%)	216 (1.3%)	41 (0.3%)	1,015 (6.3%)
AB positive	4,531 (9.7%)	118 (2.6%)	29 (0.6%)	93 (2.1%)	74 (1.6%)	16 (0.4%)	330 (7.3%)
O positive	12,781 (27.5%)	324 (2.5%)	76 (0.6%)	217 (1.7%)	187 (1.5%)	35 (0.3%)	839 (6.6%)
A negative	631 (1.4%)	17 (2.7%)	4 (0.6%)	12 (1.9%)	3 (0.5%)	1 (0.2%)	37 (5.9%)
B negative	940 (2.0%)	22 (2.3%)	6 (0.6%)	23 (2.4%)	15 (1.6%)	8 (0.9%)	74 (7.9%)
AB negative	280 (0.6%)	8 (2.9%)	1 (0.4%)	6 (2.1%)	4 (1.4%)	1 (0.4%)	20 (7.1%)
O negative	717 (1.5%)	24 (3.3%)	8 (1.1%)	14 (2.0%)	10 (1.4%)	2 (0.3%)	58 (8.1%)
Total	46,549 (100%)	1,183 (2.5%)	275 (0.6%)	815 (1.8%)	657 (1.4%)	134 (0.3%)	3,064 (6.6%)

For HIV infection, 1.1% of O-negative blood donors were found to be reactive; however, it was not significant (Fisher’s exact test = 2.98, p = 0.08). In total, 23 B-negative blood donors (2.4%) were found to be HCV reactive. This association was found to be statistically significant (χ^2^ = 4.3, p = 0.038). Overall, 148 A-positive blood donors (1.4%) were found to be syphilis reactive. This association was found to be statistically significant (χ^2^ = 3.86, p = 0.05). Moreover, 8 (0.9%) B-negative blood donors were found to be malaria reactive. This association was found to be statistically significant (Fisher’s exact test = 11.06, p = 0.001).

The total number of samples from transfusion-transmitted disease-infected blood donors collected for minor antigen typing (Fya, Fyb, S, K, k, D, C, E, c, and e) was 258 over two months, of which 123 samples were HBV reactive, 31 were HIV reactive, 38 were HCV reactive, 53 were syphilis reactive, and 23 were malaria reactive (Table 4).

**Table 4:** Antigen frequencies in some reactive blood donors. HBV: hepatitis B virus; HCV: hepatitis B virus; HIV: human immunodeficiency virus

Antigens	HBV (n = 123)	HIV (n = 31)	HCV (n = 38)	Syphilis (n = 53)	Malaria (n = 23)
Fya	107 (87.0%)	27 (87.1%)	35 (92.1%)	50 (94.3%)	18 (78.2%)
Fyb	70 (56.9%)	24 (77.4%)	20 (52.6%)	26 (49.1%)	14 (60.8%)
S	28 (23%)	10 (32.3%)	9 (24%)	11 (21%)	9 (39.1%)
K	5 (4%)	0 (0%)	2 (5%)	2 (4%)	0 (0%)
k	123 (100%)	31 (100%)	38 (100%)	53 (100%)	23 (100%)
D	113 (91.9%)	28 (90.3%)	36 (94.7%)	50 (94.3%)	20 (86.9%)
C	100 (81.3%)	27 (87.1%)	34 (89.5%)	46 (86.8%)	17 (73.9%)
E	27 (21.9%)	3 (9.7%)	7 (18.4%)	11 (20.8%)	8 (34.8%)
c	70 (56.9%)	13 (41.9%)	16 (42.1%)	31 (58.5%)	14 (60.9%)
e	123 (100%)	31 (100%)	38 (100%)	51 (96.2%)	23 (100%)

As shown in Table 4, Fyb antigen was relatively more prevalent among HIV-reactive donors (n = 24, 77.4%), while E (n = 3, 9.7%) and c (n = 13, 41.9%) antigens were relatively less prevalent compared with other infected donors. Similarly, malaria-reactive donors showed a lower prevalence of C antigen (n = 17, 73.9%) and a higher prevalence of E antigen (n = 8, 34.8%) compared with other infected blood donors.

As shown in Table 5, the most common Rh phenotype found among all transfusion-transmitted disease-infected reactive donors was CCDee: HBV (n = 53, 43%), HIV (n = 17, 55%), HCV (n = 21, 55%), syphilis (n = 22, 41.5%), and malaria (n = 9, 39%). The CcDee phenotype was relatively less prevalent in HCV (n = 7, 18%) and malaria (n = 3, 3%) reactive donors compared to other reactive donors. Among the Rh-negative infected donors, ccdee was the only phenotype observed: HBV (n = 10, 8%), HIV (n = 3, 10%), HCV (n = 2, 5%), syphilis (n = 3, 5.6%), and malaria (n = 3, 13%).

**Table 5:** Rh phenotype frequencies. HBV: hepatitis B virus; HCV: hepatitis B virus; HIV: human immunodeficiency virus

Rh phenotypes	HBV (n = 123)	HIV (n = 31)	HCV (n = 38)	Syphilis (n = 53)	Malaria (n = 23)
CCDee	53(43%)	17 (55%)	21 (55%)	22 (41.5%)	9 (39%)
CcDee	28 (23%)	8 (26%)	7 (18%)	16 (30.2%)	3 (13%)
CcDEe	18 (15%)	1 (3%)	5 (13%)	7 (13.2%)	5 (22%)
ccDEe	9 (7%)	1 (3%)	1 (3%)	2 (3.8%)	3 (13%)
ccDee	5 (4%)	0 (0%)	1 (3%)	1 (1.9%)	0 (0%)
CCDEe	0 (0%)	1 (3%)	1 (3%)	0 (0%)	0 (0%)
ccDEE	0 (0%)	0 (0%)	0 (0%)	1 (1.9%)	0 (0%)
CcDEE	0 (0%)	0 (0%)	0 (0%)	1 (1.9%)	0 (0%)
CCDEE	0 (0%)	0 (0%)	0 (0%)	0 (0%)	0 (0%)
ccdee	10 (8%)	3 (10%)	2 (5%)	3 (5.6%)	3 (13%)
Ccdee	0 (0%)	0 (0%)	0 (0%)	0 (0%)	0 (0%)
ccdEe	0 (0%)	0 (0%)	0 (0%)	0 (0%)	0 (0%)
CCdee	0 (0%)	0 (0%)	0 (0%)	0 (0%)	0 (0%)
ccdEE	0 (0%)	0 (0%)	0 (0%)	0 (0%)	0 (0%)
CcdEe	0 (0%)	0 (0%)	0 (0%)	0 (0%)	0 (0%)
CCdEe	0 (0%)	0 (0%)	0 (0%)	0 (0%)	0 (0%)
CcdEE	0 (0%)	0 (0%)	0 (0%)	0 (0%)	0 (0%)
CCdEE	0 (0%)	0 (0%)	0 (0%)	0 (0%)	0 (0%)

## Discussion

Blood, as a scarce and expensive human resource, should be prescribed judiciously and appropriately. All patients who receive regular packed red blood cell volume or any blood component are at an increased risk of TTIs. There is a risk that if someone donates blood during the window period, the tests would not detect the virus, and the donated blood could infect a blood recipient. Blood donor education, deferral of donors with risk factors for transmissible disease, and screening of donated blood by more sensitive tests aim to reduce the residual risk.

In this study, a total of 46,549 blood donors were studied for the prevalence of TTIs. In total, 3,064 (6.6%) donors were seropositive for TTIs; 1,183 (2.5%) donors were positive for HBV, 815 (1.8%) for HCV, 657 (1.4%) for syphilis, and 275 (0.6%) donors were reactive for HIV. Comparing our study with other studies performed across the country, a similar prevalence of HIV was found in Delhi (0.56%) and Kerala (0.6%) [9,10]. HBsAg and HCV reactivity were found to be higher in our study compared with the above studies. Syphilis was found to be more common in Bangalore (1.6%) [11].

Higher prevalence rates of TTIs can be multifactorial. Lack of vaccination, especially if the donors are refugees, immigration history from certain endemic zones where TTIs are more common, may be the factors for higher incidence of some diseases [12,13]. Several studies have shown that the prevalence of HIV and syphilis among replacement donors was significantly higher than that found in voluntary donors. These studies strongly indicate that replacement donors are less suitable, and there is a need to put major emphasis on encouraging voluntary donors [14-16].

Compared with the studies conducted in other parts of the world, we find a great variability in the prevalence of transfusion-transmitted diseases. HIV and HBsAg reactivity was higher than that noted in our study in most of the countries. HIV frequency among blood donors was found to be 1.5% in Italy, while in various African countries, it was 3.1% (Nigeria), 3.8% (Tanzania and Ethiopia each), and 8.5% (Mozambique). The reactivity of HBsAg was found to be very high in African countries such as Nigeria (18.6%), Mozambique (10.6%), Tanzania (8.8%), and Ethiopia (4.7%), whereas in Italy, the frequency was found to be 8.3%. Higher HCV prevalence was found among 6% of blood donors in Nigeria and 4.5% of donors in Italy. Syphilis reactivity was found to be markedly higher in the African state of Tanzania (4.7%) [12,14,17-19].

Significant associations between HIV and syphilis and syphilis and HBsAg among the blood donors have been reported in some studies. These could be because these pathogens are sexually transmitted, especially syphilis, which causes lesions that promote the transmission of HIV infection [14,20]. Furthermore, the decline in the prevalence of HIV and other sexually transmitted Infections as observed in some studies may be due to behavioural change, in particular, in the youth population [21,22].

Malaria is one of the most important public health problems in terms of morbidity and mortality in many countries around the world. In our study, we found the prevalence rate of 0.3% (134). Indian studies from various parts of the country have shown either equivalent or less prevalence of Malaria [23-26]. However, a study done by Chiodini et al. (2003) in the United Kingdom showed a slightly higher malaria prevalence rate of 0.45% [27]. They utilized a commercial enzyme-linked immunosorbent assay (ELISA) kit for the detection of antibodies to malaria and compared it with an immunofluorescent antibody test (IFAT). They concluded that ELISA was sufficiently sensitive and specific to screen at-risk donors. In contrast to the above study, Contreras et al. (1999) from Venezuela found a malaria prevalence rate of 0.8% with IFAT. Their results indicated that the IFA assay is the most reliable test to use in malaria serodiagnosis compared to ELISA [28].

The endemicity of malaria is a very complex issue that is influenced by factors related to the man-host interactions, the parasite, the vector, and the environment. Above all, malaria incidence may fluctuate according to season. Furthermore, genetic resistance to malaria has also been recognized in diseases such as sickle cell anemia, thalassemia, G6PD deficiency, and ovalocytosis. Some of the genes that confer resistance to malaria are among the most variable genes in the human genome, as seen in the human leukocyte antigen (HLA), G6PD, globin, and ABO genes. Apart from this, the Duffy null variant shows resistance to *Plasmodium* as the parasite uses the Duffy blood group antigens as a ligand to enter the red blood cells [29].

In our study, 46,549 blood donors were studied for the association of blood groups with TTIs. We found HBV and HIV infections to be most common in O-negative blood donors; however, it was statistically insignificant. We also found the B-negative blood group to be significantly associated with HCV (χ^2^ = 4.3, p = 0.038) and malaria (Fisher’s exact test = 11.06, p =0.001), and a weak association of A-positive blood group with syphilis (χ^2^ = 3.86, p = 0.05). Our study findings were consistent with another study by Tyagi et al. (2013), who found that negative blood groups were more prone to TTIs. However, in contrast to our study, they found the A-negative blood group to be more prone to HIV and HBV [30]. Our study findings are also consistent with a study by Nigam et al. (2014), who also observed that RhD-negative blood donors showed a higher percentage of seroreactivity for TTIs. Hence, they concluded that the association of TTIs with blood group and RhD types needs comprehensive large-scale studies to evaluate their association with blood group type and to categorize Rh-negative donors as high-risk donors [31]. In a study, Bharadva et al. (2016) (Gujarat, India) found A blood group (p < 0.05) to be significantly associated with HCV infection [32]. In a study by Mohammadali et al. (2014) (Tehran, Iran), the A blood group (p < 0.05) was significantly associated with HIV infection [33].

Some blood groups can act as a receptor and ligand for bacteria, parasites, and viruses. The possible pathogenesis for this susceptibility is that many organisms that may bind to polysaccharides on cells and soluble blood group antigens may block this binding [34]. In a study by Neil et al. (2004), ABO histo-blood group sugars were detected on the viral envelope protein, gp120. Thus, they concluded that incorporation of ABO antigens by HIV-1 may affect transmission of virus between individuals of discordant blood groups by interaction with host natural antibody and complement [35]. Hansen et al. (1990) showed that antibodies against blood group A and LeY inhibited HIV infection in a dose-dependent fashion, with 80% virus inhibition at micromolar concentrations (0.32-1.16 μg/50% infectious dose) [36].

The same blood group systems are shared by all human populations; the difference lies in the frequencies of their specific types. Marked variations are noted in the incidence of blood groups among different races, ethnic groups, and socioeconomic groups in different parts of the world. Knowledge of the prevalence of different blood groups is necessary for the efficient delivery of blood bank services.

In this study, transfusion-transmitted disease-infected donors were tested for Rh, Duffy, Kell, and MNS blood groups. Despite putting our maximum effort into searching the academic literature and authentic websites, we could not find any report regarding the association of minor blood groups with TTIs. However, the prevalence of minor blood groups in the non-reactive blood donor population has been studied by Makroo et al. (2013) [37]. They studied a total of 3,073 blood donors, in whom the prevalence of Fya, K, k, D, and e antigens did not show much difference compared with the reactive blood donors of our study. On the contrary, the prevalence of Fyb, S, E, C, and c antigens in the reactive donors of our study did not match the non-reactive donors of the study reported by Makroo et al.

Furthermore, the most common Rh phenotype found among all the transfusion-transmitted disease-infected reactive donors was CCDee, similar to the non-reactive donors studied by Makroo et al. Among the Rh-negative infected donors, ccdee was the only phenotype observed in our study, while Makroo et al. showed a few other phenotypes as well in their non-reactive blood donors [37].

Limitations

This study has a few limitations. The study was a single-center study. Tests for only a few minor blood group antigens were done for a short time during the study period.

## Conclusions

The knowledge of the prevalence of TTIs among the blood donors would help us understand the burden of the diseases in the population and the risks of their transmission to the patients. Voluntary donation should be encouraged for the prevention of TTIs. Replacement and voluntary donors should be screened thoroughly before blood donation, and professional donors should be deferred. Public awareness and counseling could also help curb these infections and increase blood safety. This study showed a significant association of blood groups with TTIs. Further large-scale studies are required to help us improve our blood transfusion services. The frequency of blood group antigens was different in the studied transfusion-transmitted disease-infected blood donors. The reason behind this variance needs to be further explored, and extensive studies in this field can be fruitful.
